# Impact of grazing intensities on reproduction patterns of elm trees (*Ulmus pumila*) in degraded sandy lands in China

**DOI:** 10.7717/peerj.9013

**Published:** 2020-04-16

**Authors:** Yi Tang

**Affiliations:** 1 School of Life Science, Liaoning University, Shenyang, China; 2 Institute of Statistics and Data Science, Liaoning University, Shenyang, China

**Keywords:** Duration of reproduction, Horqin Sandy Land, Reproductive allocation, Semi-arid lands

## Abstract

The effect of grazing on patterns of reproduction in trees has been little reported. We explored the effects of grazing intensities on reproductive growth, allocation patterns, and duration in elm trees (*Ulmus pumila* L.) at the Horqin Sandy Land, a degraded area in northern China. Current-year shoots were selected from branches and harvested from individual elm trees subjected to one of four grazing intensities (heavy, moderate, light, and no grazing). Shoots, flower buds, flowers, seeds, leaf buds, and leaves were collected, dried, and weighed. Results showed that the biomass in heavy, moderate and light grazing treatments is significantly higher than in no grazing treatment (*P* < 0.05). The reproductive allocation of *U. pumila* in heavy grazing treatment was significantly higher from that in the no grazing treatment (*P* < 0.05). Additionally, we found that reproduction of *U. pumila* ended later in grazed plots, suggesting the duration of reproduction is extended with grazing disturbance. Our findings suggest that *U. pumila* may prolong it s duration of reproduction and alter its reproductive biomass in response to grazing. It is not clear whether these effects are related to damage to *U. pumila* trees by grazers or whether they are due to grazers affecting soil properties or plant competitors around *U. pumila* trees.

## Introduction

Livestock grazing in sandy lands can impact vegetation and soils, leading to desertification in arid and semi-arid lands (Kraaij & Ward, 2006; Wang et al., 2015). Livestock grazing can also decrease biodiversity, reduce biomass accumulation and soil organic matter, increase soil bulk densities and alter ecosystem services (Huang et al., 2018; Tang et al., 2016; Zuo et al., 2018). Effects of grazing on plants in sandy lands are reported in seedlings, recruitment and species diversity (Lv et al., 2016; Tang, Jiang & Lv, 2014; Wang et al., 2018).

Effects of grazing on plant reproduction cause concern (Gu et al., 2017; Perez-Llorca & Vilas, 2019). Grazing could influence the reproduction of plants by altering sex ratio, reproductive biomass, and biomass allocation (Graff, Rositano & Aguiar, 2013; Niu et al., 2009; Niu et al., 2012; Ren, Zheng & Bai, 2009). Most of these studies focus on grass species.

However, effects of grazing on reproduction patterns in trees have been little reported. *Ulmus pumila* L. is the dominant tree species in sparse elm woodlands, which is the original vegetation community in the Horqin Sandy Land, one of the most extensive sandy lands in China (Tang & Li, 2018). Recently, sparse elm woodland has undergone severe desertification mainly due to human activities such as grazing (Li et al., 2003). The effects of grazing on sparse elm woodlands, especially on *U. pumila,* are of particular importance to understanding and mitigating desertification (Dulamsuren et al., 2009).

Our previous study in this region found that grazing caused an increase in seed production (Tang, Jiang & Lv, 2014). However, how the elm trees are regulating seed production in response to grazing is unknown. Limited resources are allocated among several competing functions. For *U. pumila*, more seeds produced under grazing disturbance indicates that more resources are devoted to reproduction. Therefore, we hypothesize that more biomass of *U. pumila* is allocated to reproduction in grazed lands than in lands without grazing.

Patterns of resource allocation between reproduction and vegetative growth in plants have been used to explore plant responses to environmental changes (McConnaughay & Coleman, 1999). For plants, developing adaptations to invest critical resources efficiently in response to environmental changes is very important to sustain the population (Hirayama et al., 2008; Kolb & Sperry, 1999). When reproduction and vegetative growth are simultaneous, they may trade-off in ways to maximize fitness (Worley & Harder, 1996).

*U. pumila* flowers before coming into leaf; therefore, reproduction and vegetative growth are not simultaneous. Changes in the timing of flowering could alter the allocation of resources to reproduction. How grazing treatments influence the timing of reproduction of *U. pumila* has not been reported. We hypothesize that grazing extends the duration of reproduction, which is closely related to the first hypothesis that grazing leads to an increase in reproductive allocation of *U. pumila* in sparse elm woodlands.

The objectives of this study were: (1) to determine the effects of grazing treatments on the reproductive biomass, vegetative biomass and their proportional allocation in *U. pumila* growing in sparse elm woodlands and (2) to investigate the timing of reproduction of *U. pumila* subject to grazing.

## Materials & Methods

This study was carried out in the Wulanaodu area of the Horqin Sandy Land in northern China (119°39′−120° 02′E, 42°29′−43°06′N, 480 m a.s.l.). The study area is within a semi-arid climate. The average annual temperature is 6.3 °C, with July being the warmest month averaging 23 °C, and January being the coldest month, averaging −14 °C. Mean annual precipitation is 340 mm, of which 70% falls in June, July and August. Mean annual wind velocity is 4.4 m s^−1^ and the number of gale days (>16 m s^−1^) ranges between 21 and 80. The windy season lasts from March to May, and the growing season begins in late April and ends in late September (Zhang, Wu & Tang, 2016). The prevalent wind direction is northwest, and the second most prevalent wind direction is southwest. The activity of wind erosion and sand burial on active sand dunes is most intensive in May (Yan et al., 2005).

The grazing treatments, heavy grazing (HG), moderate grazing (MG), light grazing (LG) and no grazing (CK) were established in the study region for more than 20 years. The mean stocking rates in HG, MG, and LG were 2.68 sheep units ha^−1^, 1.88 sheep units ha^−1^, and 0.76 sheep units ha^−1^, respectively (Wang et al., 2018). Over the long-term under grazing, the vegetation and soil properties are different from each other in treated plots (Tang et al., 2016). In each plot, we selected 4 adult individuals of *U. pumila* (16 individuals in all). The selected individuals were similar in size, 6–8 m in height and 15–20 cm diameter at breast height (DBH), but tree ages were not measured. The trees’ height could prevent sheep from feeding on many leaves, but sheep could seek shade under trees and feed on bark and stems.

According to previous observations, buds begin to expand at the end of March, and the seed dispersal ends mid-May. From March 25th to May 15th, 2015, we numbered and randomly selected primary branches. On each primary branch, 4 current-year shoots of similar size were selected for measurement every five days. For each current-year shoot, we collected the shoot, flower buds, flowers, seeds, leaf buds, and leaves. All collected parts were dried for 24 h at 80 °C and weighed. The vegetative growth is defined as the sum of current-year shoots, leaf buds, and leaves. Reproductive biomass is defined as the sum of flower buds, flowers, and seeds (Li et al., 2005).

We calculated reproductive allocation (RA) following the equation below:

\begin{eqnarray*}RA= \frac{R{E}_{bio}}{R{E}_{bio}+V{E}_{bio}} \times 100\text{%} \end{eqnarray*}

where RE_bio_ was the reproductive biomass, and VE_bio_ was the biomass of vegetative growth.

A generalized linear model was used to test the differences in means of response variables between four grazing treatments. The response variables were total biomass, reproductive biomass and reproductive allocation respectively. The explanatory variables were grazing treatments and observation day. For each observation day, one-way analysis of variance (ANOVA) was used to test for differences in reproductive allocation between four treatments. Levene’s test was used to test the homogeneity of variance. If the variance satisfied the homogeneity test, a Turkey HSD test was used for multiple comparisons. If the variance failed the homogeneity test, then a Kruskal–Wallis test and Nemenyi test were used to compare treatments, adjusting for multiple comparisons. The reproductive allocation data were arcsine square root transformed before ANOVA analysis. We conducted all tests using R software (R Core Team, 2015). Differences were considered to be significant when the *P*-value was <0.05.

## Results

Average biomass and reproductive biomass across the observation period both varied significantly among grazing treatments (Table 1). Average reproductive allocation across the observation period differed only between heavy grazing and all other treatments, while all other treatments did not differ significantly (Table 1).

**Table 1:** Results of generalized linear models, where biomass, reproductive biomass and reproductive allocation work as response variables, respectively.

	Biomass				Reproductive Biomass				Reproductive allocation			
	Estimate	SE	*t*-value	*P*-value	Estimate	SE	*t*-value	*P*-value	Estimate	SE	*t*-value	*P*-value
Intercept	0.32	0.13	2.57	0.01	0.13	0.12	1.13	0.26	0.17	0.13	1.32	0.19
LG	0.45	0.09	4.71	<0.001	0.27	0.09	3.08	0.00	0.02	0.11	0.22	0.82
MG	0.40	0.09	4.23	<0.001	0.22	0.09	2.47	0.01	−0.03	0.11	−0.32	0.75
HG	0.25	0.09	2.68	0.01	0.32	0.09	3.55	0.00	0.22	0.11	2.09	0.04
Mar.30	0.12	0.16	0.77	0.44	0.07	0.15	0.47	0.64	0.07	0.16	0.45	0.66
Apr.5	0.08	0.16	0.49	0.62	0.02	0.15	0.16	0.87	−0.06	0.16	−0.40	0.69
Apr.10	0.12	0.16	0.78	0.44	0.09	0.15	0.60	0.55	0.13	0.16	0.81	0.42
Apr.15	0.74	0.16	4.68	<0.001	0.64	0.15	4.30	<0.001	0.66	0.16	4.00	<0.001
Apr.20	0.79	0.16	5.00	<0.001	0.70	0.15	4.71	<0.001	0.76	0.17	4.59	<0.001
Apr.25	1.06	0.16	6.70	<0.001	0.96	0.15	6.51	<0.001	1.02	0.17	5.91	<0.001
Apr.30	1.59	0.16	10.12	<0.001	1.50	0.15	10.17	<0.001	1.39	0.18	7.54	<0.001
May.5	1.85	0.16	11.77	<0.001	1.63	0.15	11.06	<0.001	1.15	0.18	6.52	<0.001
May.10	1.79	0.16	11.37	<0.001	0.43	0.15	2.88	0.00	1.29	0.18	7.14	<0.001
May.15	0.34	0.16	2.18	0.03	−0.13	0.15	−0.86	0.39	−1.77	0.18	−9.73	<0.001

**Figure 1:**
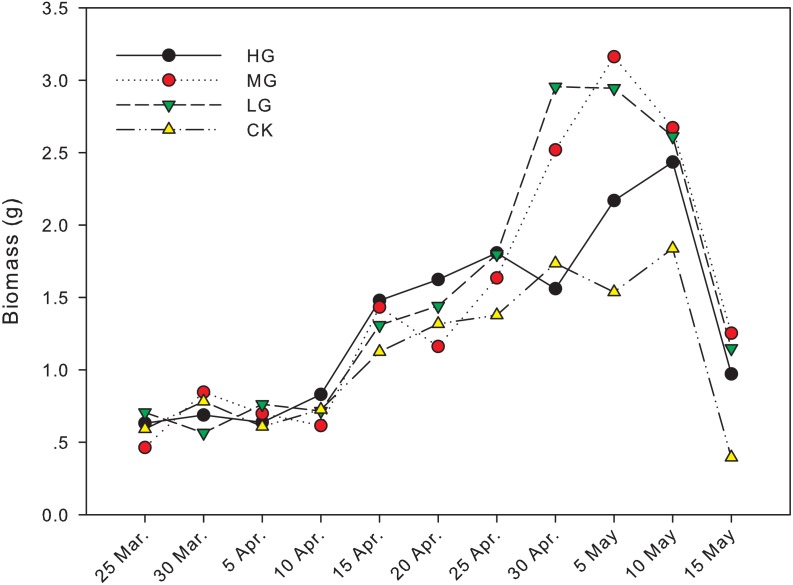
Changes in biomass of *U. pumila* by grazing intensities.

The difference in biomass between grazing and no grazing treatments is significant from April 30th to May 15th, except on May 10th (Fig. 1). On April 30th, the biomass in light grazing plots was significantly higher than that in heavy grazing and no grazing treatments (*P*  < 0.05). On May 5th, the biomass in moderate and light grazing plots was 3.16 and 2.94 g, respectively, and significantly higher than that in heavy grazing plots (2.17 g, *P* < 0.05). Meanwhile, the biomass in heavy grazing plots was significantly higher than that in no grazing plots (*P* < 0.05). On May 15th, biomass under moderate grazing was significantly higher than in plots without grazing (*P* < 0.05, Table 1, Fig. 1).

**Figure 2:**
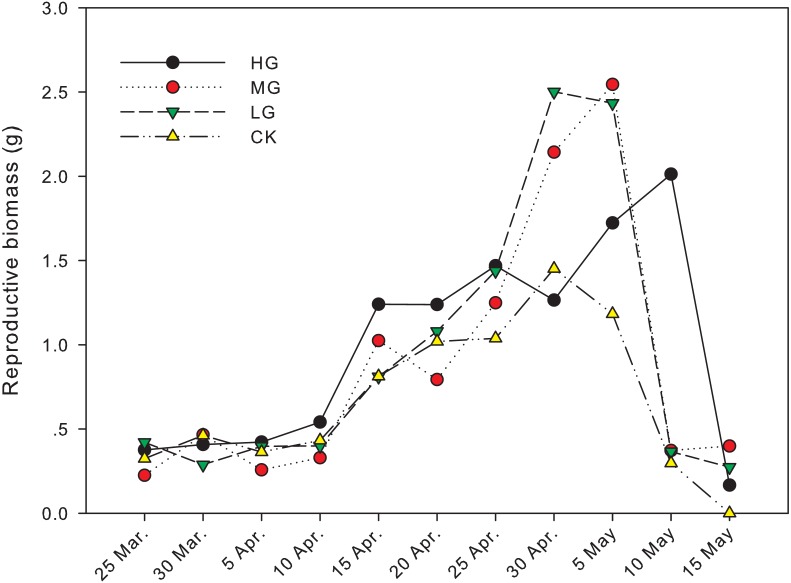
Change in reproductive biomass of *U. pumila* by grazing intensities.

The difference in reproductive biomass between grazed treatments and no grazing treatment was significant from April 30th to May 15th. On April 30th, the reproductive biomass in light grazing plots was 2.50 g and significantly higher than that in heavy grazing and no grazing plots (1.26 g and 1.45 g, respectively, *P* < 0.05). On May 5th, the reproductive biomass in moderate and light grazing plots was 2.55 and 2.43 g, respectively, and significantly higher than that in heavy grazing and no grazing plots (1.72 g and 1.18 g, respectively, *P*  < 0.05). On May 10th, reproductive biomass under heavy grazing was significantly higher than that in the other three treatments (*P* < 0.05). On May 15th, reproductive biomass under moderate grazing was significantly higher than in plots with no grazing (*P* < 0.05, Table 1, Fig. 2).

**Figure 3:**
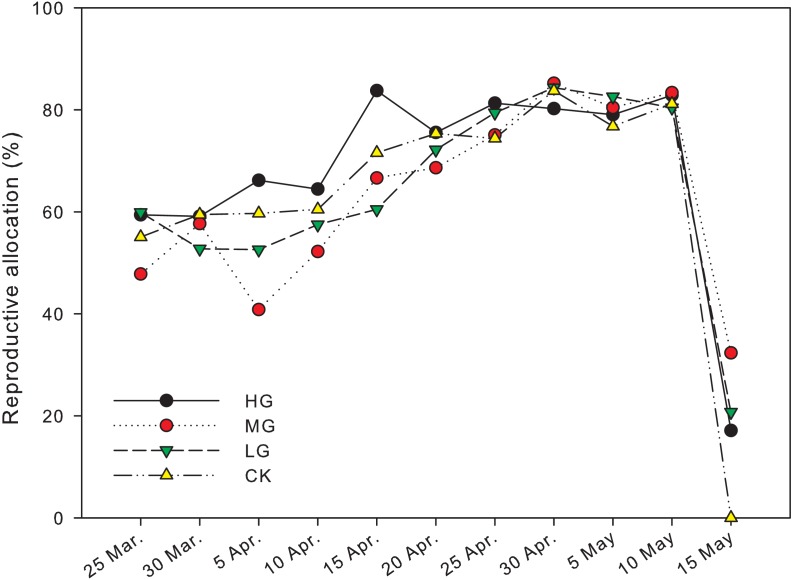
Changes in reproductive allocation of *U pumila* by grazing intensities.

The reproductive biomass allocation under grazing treatments was significantly different from that in plots with no grazing on April 5th andMay 15th (*P* < 0.05). The reproductive biomass allocation in a plot with moderate grazing was 40.8 ± 13.4% on April 5th and significantly lower than heavy and no grazing plots, where the reproductive biomass allocation was 66.2 ± 2.9% and 59.7 ± 1.9% respectively (*P* < 0.05). The reproductive biomass allocation with heavy grazing was 83.8 ± 0.6% on April 15th and significantly higher than light grazing plots, where the reproductive biomass allocation was 60.5 ± 4.5% (*P*  < 0.05). The reproductive biomass allocation in moderate grazing plots on May 15th was 32.3 ± 12.1%, which was significantly higher than that in plots with no grazing, where the reproductive biomass allocation was 0% (*P* < 0.05). In control plots, the end of reproduction was earlier than May 15th, and was earlier than the end of reproduction period in the grazed plots (Fig. 3, Table 1).

## Discussion

Reproductive allocation in the no grazing plots ended before May 15th, which was earlier than all grazed plots. supporting our hypothesis that grazing treatment could extend the duration of reproduction in *U. pumila* in the Horqin Sandy Land. The longer duration of reproduction could help *U. pumila* produces more seeds, which might allow more opportunity for regeneration.

This result could partly explain why the total number of seeds of *U. pumila* in grazing plots was previously found to be significantly higher than that in control plots (Tang, Jiang & Lv, 2014). *U. pumila* might regulate its reproduction duration in response to grazing treatments,suggesting a complex mechanisms to regulate seed production.

Significant differences in biomass and reproductive biomass of *U. pumila* were observed between grazed and control plots, which supported the hypothesis that grazing led to an increase in biomass and reproductive biomass. It suggests that the response of *U. pumila* seems to be related to grazing intensities, with later season peaks in reproductive allocation observed with heavier grazing intensity (Fig. 3).

Grazing disturbance is one of the significant threats to land degradation especially in arid or semi-arid ecosystems (Lu et al., 2015). The effects of grazing on reproductive biomass allocation in grasses and shrubs, but not trees are reported in previous literature. For example, Hickman and Hartnett found that grazing decreased the reproductive biomass allocation in *Amorpha canescens* Pursh, a tall perennial grass (Hickman & Hartnett, 2002). In contrast, Gao, Gao & He (2014) reported that grazing increased the reproductive biomass allocation in *Stipa grandis* P.A. Smirn, a perennial tussock grass. Our study provides an example of how trees respond to grazing disturbance. Our results showed that the biomass, reproductive biomass, reproductive allocation, and reproductive duration can increase in grazing plots. This is not fully consistent with either of the grass examples above. Compared with grasses, the *U. pumila* is relatively taller, and much of the plant may be out of reach for sheep in the grazing plots. In addition to responses to herbivory, *U. pumila* might be responding to differences in soil conditions or reduced grass competition in grazing treatments (Verwijmeren et al., 2019).

The difference in soil conditions or species competition might influence the reproduction pattern in *U. pumila* through indirect ways, for instance, altering the nutrients supply (Shen et al., 2019). It seems that it is necensary to clarify the direct or indirect effects of grazing around *U. pumila* in further studies. Besides, effects of grazing on *U. pumila* seedlings are not studied here but it has been previously reported that seedling densities of *U. pumila* are not significantly influenced by grazing (Tang, Jiang & Lv, 2014). It seems that effects of grazing on *U. pumila* are different in reproduction and seedling stages.

In this study, the biomass, reproductive biomass and reproductive allocation of *U. pumila* in response to grazing seemed to change towards the end of the reproductive season (April 30th to May 15th). This might indicate that *U. pumila* regulates reproductive and vegetative biomass at a particular time point. The mechanism(s) involved in regulation of reproductive responses could be explored with transcriptome analysis.

## Conclusions

Our study indicates that: (1) *U. pumila* extends the duration of reproduction under grazing; (2) *U. pumila* changes biomass and reproductive biomass to response to grazing; and (3) *U. pumila* changes reproductive allocation under heavy grazing treatment. These results suggest that some aspects of *U. pumila’s* reproduction might benefit from grazing treatments, but it is not clear whether these effects are due to direct or indirect effects of grazing around *U. pumila*. Furthermore, effects of grazing on *U. pumila* seedlings were not studied and might be different from effects on mature trees examined here.

##  Supplemental Information

10.7717/peerj.9013/supp-1 Supplemental Information 1 Raw data

## Additional Information and Declarations

### Data Availability

The following information was supplied regarding data availability: The raw measurements are available as a Supplementary File.
